# Hydropneumothorax With Persistent Air Leak in a Patient With Mild COVID-19 Disease

**DOI:** 10.7759/cureus.22150

**Published:** 2022-02-12

**Authors:** Ioannis N Pantazopoulos, Athanasios Pagonis, Garifallia Perlepe, Christos F Kampolis, Konstantinos I Gourgoulianis

**Affiliations:** 1 Emergency Medicine, University General Hospital of Larissa, Larissa, GRC; 2 Respiratory Medicine, University General Hospital of Larissa, Larissa, GRC; 3 Emergency Medicine, "Hippokration" General Hospital of Athens, Athens, GRC

**Keywords:** pleurodesis, bullectomy, persistent air leak, pneumothorax, pleural effusion, hydropneumothorax, covid-19

## Abstract

COVID-19 is a pandemic viral disease with a catastrophic global impact. The severity of COVID-19 symptoms ranges from very mild to severe and affects mainly the respiratory system. Spontaneous pneumothorax and pleural effusion are rarely seen in spontaneously breathing COVID-19 patients. We herein report a case of a patient with mild COVID-19 disease presenting to the emergency department with hydropneumothorax. Due to persistent air leak, the patient was managed with video-assisted thoracoscopic surgery (VATS) bullectomy and talc pleurodesis. Clinicians managing these patients should be alert to early diagnose this complication.

## Introduction

Coronavirus disease 2019 (COVID-19), caused by severe acute respiratory syndrome coronavirus 2 (SARS-CoV-2), broke out in Wuhan, China, in December 2019 and became a pandemic within months. Since then it has affected more than 364 million people worldwide. The severity of COVID-19 symptoms ranges from very mild to severe and affects mainly the respiratory system [1]. Non-respiratory complications of COVID-19 have included cardiac injury (mainly acute heart failure and myocarditis), arrhythmia, septic shock, liver dysfunction, acute kidney injury, coagulopathy, neurologic manifestations (including impaired consciousness and stroke), and multi-organ failure [2]. Spontaneous pneumothorax and pleural effusion were rarely seen in COVID-19 patients. It has been reported that patients with pleural effusion might have severe inflammation and a poor prognosis [3]. On the other hand, pneumothorax is thought to be related to the structural changes that occur in the lung parenchyma mainly in patients with severe acute respiratory distress syndrome (ARDS) [4].

We describe the case of a spontaneously breathing patient with mild SARS-CoV-2 disease confirmed by real-time polymerase chain reaction (RT-PCR) that presented to the emergency department (ED) with hydropneumothorax.

## Case presentation

A 23-year-old Caucasian patient with no medical history presented to the emergency department (ED) complaining of fever after being in contact with a COVID-19 positive patient. His medical history revealed an influenza-like syndrome starting five days ago with a fever up to 38.6 °C, dry cough, and headache.

On admission, the patient was alert, with a respiratory rate of 22 breaths/min, pulse oximetry at 98% on room air, heart rate at 78 beats per minute, and blood pressure at 118/63 mmHg, with no evidence of right or left heart failure. Lung auscultation found diminished lung sounds at the right hemithorax. Blood gas analysis revealed pH: 7.43, PaO2: 79 mmHg, and PaCO2: 39 mmHg. On general examination, no lymphadenopathy was observed, and other systems, including cardiac and abdominal examination, were normal. Lab tests were unremarkable. Nasopharyngeal swab RT-PCR testing for COVID-19 was positive (cycle threshold: 19).

**Figure 1:**
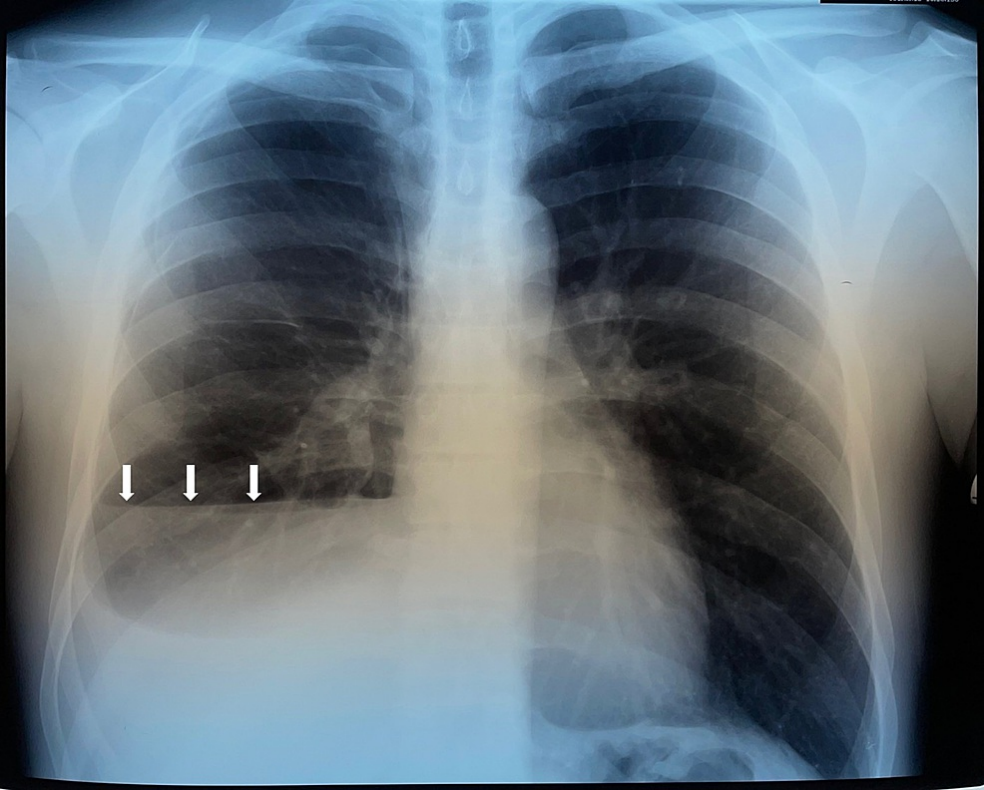
Initial chest x-ray showing air fluid level in the right thoracic cavity (white arrows)

**Figure 2:**
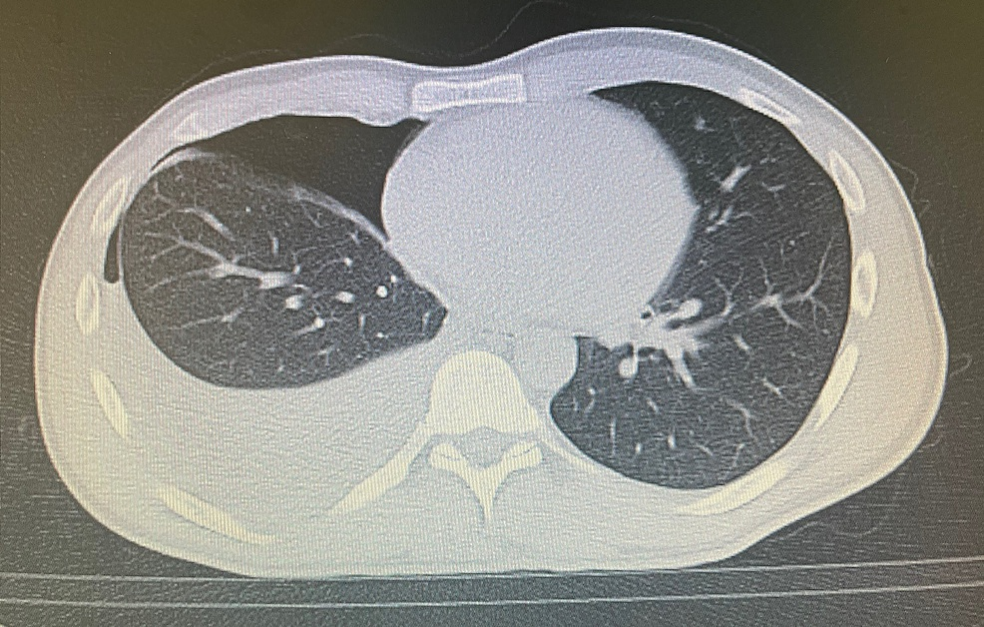
Chest computed tomography showing a large right-sided hydropneumothorax

An upright chest x-ray showed an air-fluid level in the right hemithorax (Figure 1). Chest computed tomography showed a collapse of the right lung, a large effusion, and pneumothorax (Figure 2) with normal lung parenchyma. No ground glass opacities, consolidations, bronchopleural fistula, pneumomediastinum, or subcutaneous emphysema were observed.

The patient was admitted to the pulmonology ward after a chest tube was inserted on the right hemithorax, draining a semi-clear, yellow fluid and air. The chest tube was initially placed on -20 cm H2O suction. Pleural fluid was sent for cell count, biochemistry, cytology, acid fast bacilli staining, and Gram-stain, culture, and sensitivity. 

Diagnostic pleural analysis revealed an exudative effusion with pleural fluid Ph of 7.31, the glucose of 1 mg/dL, and very high lactate dehydrogenase (LDH) of 1381 U/L. The pleural fluid differential white blood cell count had 20% lymphocytes, 32% neutrophils, 48% atypical mononuclear cells with cytology negative for malignant cells. Adenosine deaminase of the pleural fluid was 128 IU/L. Pleural fluid stains and culture were negative for bacteria and tuberculosis. Routine screening tests for connective-tissue disease were also negative. The interferon-gamma release assay (IGRA) for tuberculosis (TB) result was negative. SARS-Cov-2 performed by RT-PCR (Direct SARS-CoV-2 Real-Time PCR kit, Vircell, Granada, Spain) was not detected in pleural fluid. 

**Figure 3:**
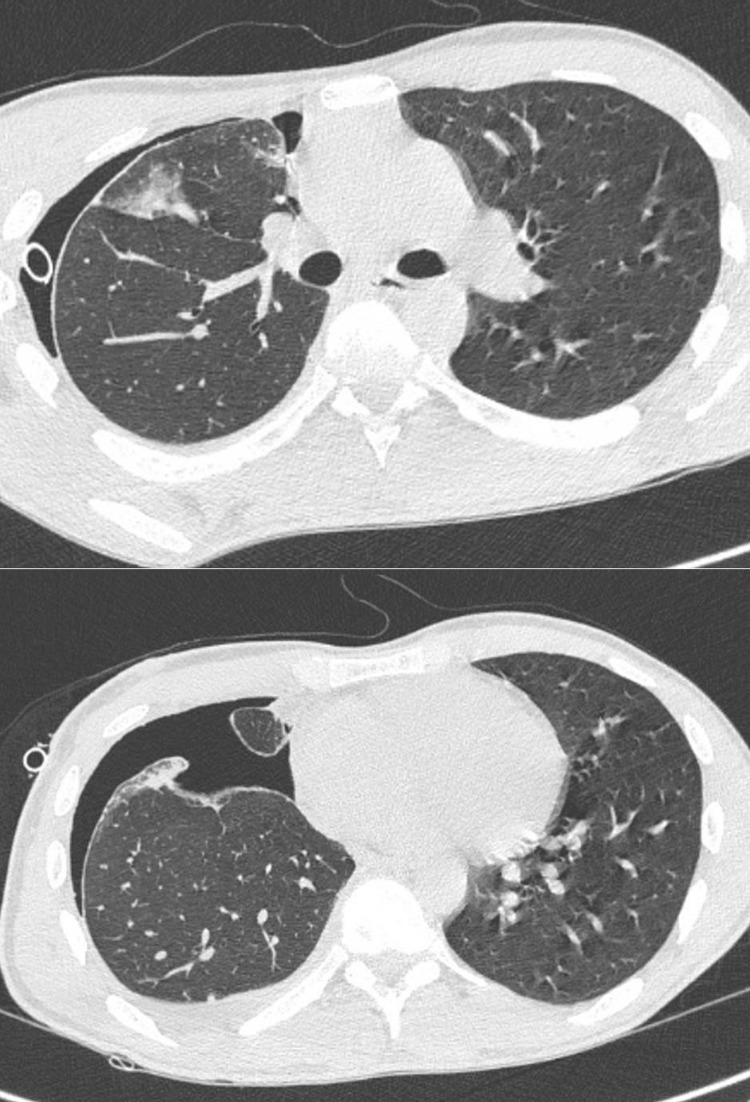
Chest computed tomography performed 10 days following chest tube insertion showing non-expandable right lung

The patient was maintained on antibiotics (ceftriaxone), and analgesia (paracetamol). Both the effusion and air persisted for 10 days (Figure 3) and the patient was transferred to the department of thoracic surgery. Three days later due to the persistent air leak, the patient was treated surgically with video-assisted thoracoscopic surgery (VATS) bullectomy and talc pleurodesis. Pleural biopsies excluded TB. The evolution was favorable without any complication and the patient was finally discharged. No recurrence was observed six months after hospital discharge. Written consent was obtained from the patient for publication. 

## Discussion

To our knowledge, this is the first case of hydropneumothorax in a spontaneously breathing patient with mild COVID-19 disease. Pleural effusion is an uncommon finding in coronavirus infection and has been more commonly reported in critically ill patients with Multisystem Inflammatory Syndrome (MIS) [5]. Interestingly, our patient had normal lung parenchyma and normal oxygen level with no evidence of MIS. Moreover, in most cases, pleural effusion appears 11 days after onset of COVID-19 symptoms [5], while in our case pleural effusion was identified five days after symptoms onset. On the other hand, in the majority of cases, the pleural effusion has been reported to be unilateral, as in our case. Furthermore, the characteristic findings of pleural fluid in COVID-19 patients are exudative, lymphocytic, or neutrophilic-predominant pleural fluid with remarkably elevated lactate dehydrogenase (LDH) levels, as in our case [5]. The pathophysiological mechanism is not clear, however, it seems that invasion of the lung by SARS-CoV-2 with subsequent inflammation causes alveolar and endothelial damage, which results in the accumulation of interstitial fluid due to leaky microvasculature. Excess interstitial fluid may enter the pleural space driven by interstitial-pleural pressure gradient [6]. Furthermore, an increase in permeability of the pleura caused by direct invasion by the SARS-CoV-2, subsequent cytokine storm, and inflammation of the visceral pleura may enhance fluid accumulation [6]. Regardless of the pathophysiological mechanism, it has been reported that pleural effusion may represent an adverse prognostic sign indicating bacterial super-infection, although this was not confirmed in our patient [5]. 

COVID-19 infection leading to acute respiratory distress syndrome might be associated with a spontaneous air leak from ruptured alveoli [7]. Released alveolar air may travel via peribronchovascular sheaths into the mediastinum, the pleural space, or the subcutaneous tissue. The diffuse alveolar injury in severe COVID-19 pneumonia results in fragile alveoli that are prone to rupture [7]. Moreover, ventilation with positive airway pressure increases the chances of air leaks. However, primary spontaneous pneumothorax in spontaneously breathing patients with mild COVID-19 disease is extremely rare. A possible mechanism of pneumothorax in our case is bulla formation. It has been demonstrated that irrespective of the stage of the disease alveolar damage in COVID-19 patients may cause the development of bulla [8]. Necrosis due to ischemia caused by microvascular damage, remodeling of interstitial matrix, and bronchial obstruction due to exudates with distal hyperinflation due to a check-valve mechanism have been proposed as possible mechanisms for bulla formation [9]. Moreover, persistent coughing with a subsequent increase in intrathoracic pressure can lead to an alveolar rupture in the presence of an underline bulla [10]. 

 Many studies have correlated tuberculosis with COVID-19. People with tuberculosis have a higher chance of getting infected with COVD-19, but pre‐existing tuberculosis can lead to severe complications from COVID‐19 [11]. In our case, only the high ADA level was consistent with TB, but IGRA test, pleural fluid stains and culture, and pleural biopsies were negative. 

Patients with hydropneumothorax are commonly managed conservatively with chest drain and observation. However, in case of persistent air leak, more invasive techniques, such as VATS or thoracotomy and medical pleurodesis, are needed [12]. In our case, we successfully performed VATS bullectomy with talk pleurodesis. Six months after hospital discharge, the patient did not present any complications. 

## Conclusions

Hydropneumothorax rarely presents as the first manifestation of COVID-19 in young, spontaneously breathing patients. Clinicians managing these patients should be alert to early diagnose this complication. Persistence of symptoms and/or radiological findings may warrant surgical intervention.
